# Retrospective Study of Salinomycin Toxicosis in 66 Cats

**DOI:** 10.4061/2010/147142

**Published:** 2010-03-22

**Authors:** Akos Pakozdy, Iris Challande-Kathman, Marcus Doherr, Sigitas Cizinauskas, Simon J. Wheeler, Anna Oevermann, Andre Jaggy

**Affiliations:** ^1^Department for Companion Animals and Horses, Small Animal Clinic, University of Veterinary Medicine Vienna, 1210 Vienna, Veterinaerplatz 1, Austria; ^2^Division of Animal Neurology, Department of Clinical Veterinary Medicine, Vetsuisse Faculty, University of Bern, Bremgartenstrasse 109a, 3012 Bern, Switzerland; ^3^Small Animal Hospital AISTI, Vantaa, Finland; ^4^Department of Clinical Veterinary Sciences, University of Helsinki, Virtatie 9, 01600 Vantaa, Finland; ^5^Department of Clinical Sciences, College of Veterinary Medicine, North Carolina State University, 4700 Hillsborough Street, Raleigh, NC 27606, USA

## Abstract

We examined 66 cats with salinomycin intoxication. Salinomycin caused different LMN signs of varying degrees of severity in all cases. Changes in blood work were unspecific, with the most frequent being increased serum creatine kinase activity, leukocytosis, and increased liver enzymes. Pathological electrodiagnostic findings: fibrillation potentials and positive sharp waves were detected in 10 cases, motor nerve conductance velocity was mildly decreased in 8/12 cats, and sensory nerve conductance velocity and repetitive nerve stimulation were normal in all examined cases. In five cases the peripheral neuropathy was confirmed by pathohistology. Fluid therapy and supportive care were used as therapy and 52 cats recovered completely. The probability for complete remission was significantly different between mildly and severely affected cases. It seems that the severity of clinical signs and prognosis correlate well with the amount of toxin ingested. We conclude that early recognition and decontamination combined with supportive care results in complete recovery.

## 1. Introduction

In spring 1996 there was an outbreak of salinomycin-related neurological dysfunction in cats in the Netherlands and Switzerland. Epidemiological information on the affected cats indicated that this outbreak was related to the feeding of two brands of dry food from one manufacturer and was caused by contamination of the food with the coccidiostatic drug salinomycin. The clinical signs ranged from mild paraparesis to severe tetraparesis with autonomic dysfunction.

Salinomycin is a monovalent ionophore and is used as a coccidiostatic drug in poultry and as a growth promoter in chickens, pigs, and cattle. Ionophores can cause toxicosis in nontarget species, however, it has been reported in sheep [1], pigs [2], horses [3, 4], dogs [5], and cats [6]. Salinomycin forms a lipid-soluble complex with cations and can cause loss of intracellular potassium, which results in ATP inhibition, cell energy depletion and consequently cell death [7]. The clinicopathological changes most often reported include RBC fragility, electrolyte disturbances, CK elevation, and myoglobinuria. Pathological changes are found in the myocardium, skeletal muscle, and peripheral nerves [8]. Although the toxicity of salinomycin is well known, there are no detailed studies on clinical, neurological, laboratory and electrophysiological findings in cats. The aim of this study was to describe retrospectively the historical, clinical, laboratory, and electrophysiological data as well as outcome in cats with salinomycin intoxication.

## 2. Material and Methods

Sixty-six well-documented medical records of cats with clinical signs of polyneuropathy caused by the presence of salinomycin in two different brands of commercial cat food (Food I and Food II) were selected and reviewed. The study population included 54 cases reported by referral vets and 12 cases admitted to the Small Animal Teaching Hospital of the University of Bern during May 1997. All cats were subjected to a complete physical and neurological examination. The cats were classified into six groups according to their neurological status (Table 1). In two thirds of the cases additional laboratory investigations including complete blood cell counts (CBC), blood chemistry, electrolyte evaluation, as well as urinalysis were performed. Cerebrospinal fluid (CSF) analysis (*n* = 13) included total white cell count, cytomorphology, protein determination, and Pandy reaction. In selected cases, clinical electrophysiological tests were carried out including electromyography (EMG), motor nerve conduction velocity (MNCV), sensory nerve conduction velocity (SNCV), and repetitive nerve stimulation testing (RNS). Two cats had serial EMG and NCV tests to follow the course of the disease. Clinical electrophysiological tests were performed under general anaesthesia. Cats were premedicated with medetomidine (Domitor, Orion Corp. Farmos, Turku, SF; 0.025 mg/kg body weight (BW)) and anaesthesia was induced with intravenous bolus of propofol (Disoprivan, Zeneca Ltd., Macclesfield, Cheshire, UK; 1 mg/kg BW, IV) until effect. The cats were then intubated and oxygen was administered. Finally, medetomidine was antagonized with atipamezol (Antisedan, Orion Corp. Farmos, Turku, SF; 0.0625 mg/kg BW, IV). Clinical electrophysiological tests were recorded using two-channel equipment (Dantec Cantata; Dantec). Stimulation and recording cables (Dantec 13 PO2/12 and Dantec 13 L01, respectively). and recording and stimulation electrodes (Dantec 9013 L0492 and Monopolar 13 R 11 Dantec. respectively) were used as described previously [9]. The MNCV of the left peroneal nerve between the trochanter major and the popliteal fossa was assessed by the onset-latency method. The recording concentric needle was inserted into the motor point of the cranial tibial muscle and two pairs of stimulating electrodes were placed perpendicularly to the peroneal nerve in the trochanteric and popliteal fossa [10]. The position of the stimulating electrodes was optimized until Compound Motor Action Potential (CMAP) with maximal amplitude was obtained at 1.5-to 2.5-milliampere (mA) stimulation current. The SNCV and RNS of the left peroneal nerve were assessed as described by Redding et al. [11].

Peroneal nerve (*n* = 7) and cranial tibial muscle biopsies were performed in selected cases (*n* = 7) and a postmortem examination was available in five cats.

In order to compare different diagnostic parameters between clinical groups, the initial categories (Groups) 1 and 2 and 3–5 were combined in order to increase the number of animals in the comparison groups. Animals in Group 6 were omitted since they belonged to another clinical entity. Subsequently a Fisher's Exact Test (2-sided) was used to statistically assess the association between the clinical score and other disease-related parameters.

## 3. Results

**Figure 1:**
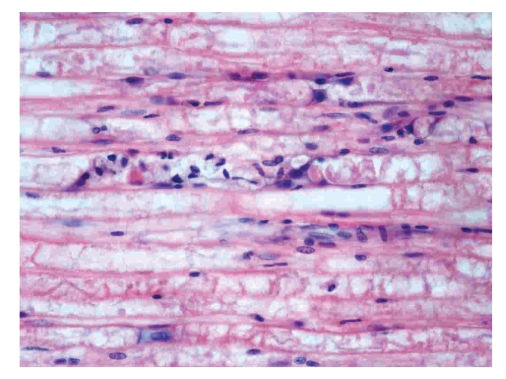
Peripheral nerve, cat. Wallerian type degeneration characterized by digestion chambers containing fragmented axons, degenerated myelin and numerous foamy macrophages. Schwann cells are swollen. Hematoxylin & Eosin, 400×.

Thirty-two male and 34 female cats were included, ranging in age from 6 months to 11 years. Sixty-four European shorthaired cats, one Siamese, and one Manx cat were studied. Lower motor neuron (LMN) disease was present in all cases. The neurological examination showed LMN paraparesis (Group 1, *n* = 19), LMN paraplegia (Group 2, *n* = 23), LMN tetraparesis (Group 3, *n* = 11), LMN tetraplegia (Group 4, *n* = 5), LMN tetraplegia, cranial nerve deficits with dyspnoea (Group 5, *n* = 5), and vestibular syndrome with autonomic signs (Group 6, *n* = 3) (Table 1). Acute onset (1–3 days duration) was documented in 50 cases and subacute onset (more than 3 days) in 16 cases. The chronic cases had a higher probability of developing more severe clinical signs (Table 4). Blood work was abnormal in 20/42 cases. The most common change was increased serum creatine kinase (CK) activity (*n* = 17), leukocytosis with mild left shift (*n* = 11), and increased liver enzymes (*n* = 9). Cats in the more severely affected groups showed more frequent changes in the serum biochemistry (9/17 in Groups 3–5) than those in less severely affected groups (5/23 in Groups 1 and 2). This difference was significant (*P* = .0011) (Table 4). Electromyography was carried out in 16 cats. Pathological findings such as fibrillation potentials and positive sharp waves were detected in 10 cases. Extensor and flexor muscles were involved with equal distribution. Proximal muscles were more affected than distal muscles, and hind limbs (bilateral) more than fore limbs. The MNCV was 70 m/s so interpreted as mildly decreased in 8/12 cats [12], the SNCV was normal in all 8 cats examined, and RNS was normal in all 7 examined cats (Table 3). The EMG and MNCV were repeated in 2 cases with similar finding to the previous. The electrophysiological changes were significantly more frequent in Groups 3–5 than in Groups 1 and 2 (Table 4). Nerve biopsy was taken in seven cases and revealed a peripheral neuropathy in five cats (Figure 1). Axonal swelling, fragmentation, axonal loss, and digestion chamber formation were consistent with salinomycin toxicity, as described previously by Van der Linde-Sipman et al. [6] (Table 2.). The peripheral neuropathy was confirmed by biopsy only in Groups 3–5. The CSF analysis was available in Groups 1–5 and was unremarkable in all 13 examined cases. The mean duration of feeding on food containing salinomycin was 4.5 to 6 days in all groups. The majority of cases (64/66) were exclusively fed with this food. The daily intake of food was different and ranged between 1/6 and 1 1/2 tins (Table 1). As salinomycin does not have a specific antidote, the therapy was fluid therapy (Lactated Ringer Solution, two maintenance doses) and supportive care. Urination was accomplished by manual expression of the bladder as necessary; a faecal softener was used if constipation occurred. Soft bedding and frequent position changes were performed in recumbent cats. Daily physiotherapy included massage, passive joint-movement, and gait exercise, as described previously by Jaggy and Kathmann [13]. The duration of recovery was different between the groups. The number of cats with complete recovery was 52. All cases (*n* = 5) that did not survive were severely ill and belonged to Group 5. Nine cats had persistent neurological signs. The probability for complete remission was significantly different between Groups 1 and 2 and Groups 3–5 (*P* = .0002) (Table 4). The recovery time was different between the groups with a marked contrast between the mild (Groups 1 and 2) and severe (Groups 3–5) cases. The clinical outcome is summarized in Table 3.

## 4. Discussion

We examined 66 cats with salinomycin intoxication. Salinomycin caused different LMN signs of varying degrees of severity in all cases. There were no clinical signs of CNS involvement or abnormal CNS findings on pathological examination in our cats, a similar finding to that reported previously in horses [4].

It has been shown that high doses of salinomycin can cause death within minutes. Clinical signs usually develop within a few hours to days after ingestion [4, 5, 8]. This was consistent with our observations. Most cases (50/66) had an acute onset and became abnormal within three days. The LMN signs in the present study are not surprising in the light of a previous report in which peripheral neuropathy characterized by primary axonal degeneration and secondary degeneration of the myelin sheath was confirmed [6]. The clinical signs, which varied from paraparesis to tetraplegia, were bilateral, symmetrical, and progressed from the pelvic to thoracic limbs. In some cats (*n* = 5, Group 5) cranial nerves were affected and some cats were dyspnoeic. Interestingly, dyspnoea was observed only in combination with severe tetraplegia. Respiratory problems were found in 7/10 dogs with ionophore intoxication [5] (Safran et al. 1993) and were reported to be the main cause of death or euthanasia in cats with salinomycin intoxication [6].

Leucocytosis was observed in 12/42 cats and was considered to be stress-related, resembling previous findings in horses [4]. The observed CK elevation (17 cats) suggests enzyme leakage from secondary damaged muscle fibrils and is most likely to originate from skeletal muscle and myocardium. Myocardial degeneration was observed in all cats; focal hyaline degeneration of the skeletal musculature was previously seen occasionally in cats with salinomycin intoxication [6]. Mildly increased liver enzymes were thought to be due to unspecific signs of intoxication as ionophores are metabolized by the liver and the liver is the primary site of ionophore storage. Increased liver enzymes and myoglobinuria have been detected in horses and humans with ionophore poisoning [14]. However, the latter was not found in our cases. The small number of cases subject to electrophysiological testing does not allow objective conclusions but it seems to be a tendency that in more severe (and chronic) cases more pronounced electrodiagnostic changes were recorded. They were mainly seen as denervation potentials (EMG changes) and mildly decreased MNCV which suggest more axonal involvement. Salinomycin chelates monovalent cations which facilitates cation cell membrane transport influence the electrical potential and energy metabolism which is the basic of toxicologic effect [8]. This may primarily affects axons and only secondarily Schwann cells.

These changes were significantly more frequent in Groups 3–5 than in Groups 1 and 2 (Tables 2 and 4). The SNCV was normal in all examined cases, which suggests that the motor part of the nerve (motor neuropathy) was mainly affected by intoxication. This finding contradicts a previous report in which both sensory and motor nerves were reported to be affected [6]. The normal RNS in all cases suggests that the function of the neuromuscular junction was not impaired. The nerve biopsy diagnosed peripheral neuropathy in all cases of Groups 3–5 but it was normal in Groups 1 and 2. However, the small number of cats examined hinders more solid conclusions. The lack of pathological changes in more mildly affected cases may be related to functional nerve conduction disturbance with mild or no morphological changes [8]. The prognosis in animals with salinomycin poisoning varies. It seems that there is a marked difference of susceptibility to ionophores between species. Dogs and horses may be more sensitive than other animals [5]. The majority (79%) of affected cats recovered with supportive care and physiotherapy. Among the severely affected cats (Groups 3–5) the recovery rate was significantly lower and the recovery time longer than in Groups 1 and 2 (Table 8). All five cats in Group 5 died.No lethal cases were observed in the other groups. It seems that tetraplegia and impairment of cranial nerves and dyspnoea are connected with a grave prognosis in cats with salinomycin intoxication. On the other hand, tetraplegic animals in which the cranial nerves and breathing function were not impaired all recovered. Artificial ventilation may have been of therapeutic benefit in cats with dyspnoea as the peripheral neuropathy impaired the function of the respiratory muscles. Intermittent positive pressure ventilation was reported to be successful in dogs. In the respective report, all 10 affected dogs survived and showed full recovery. The recovery time takes 2–50 days depending on the severity of the case [5]. 

In conclusion, we have reported detailed clinical–pathological data of salinomycin intoxication in cats and provided useful information on the prognosis of affected animals. It seems that the severity of clinical signs and prognosis correlate well with the amount of toxin ingested, although individual variations might be present. The aim of this paper is also to draw clinicians attention to the danger of ionophores (salinomycin, monensin, and lasalocid) and to include this toxic polyneuropathy in the list of possible differential diagnoses in cats with LMN tetraparesis or tetraplegia.

## Figures and Tables

**Table 1:** Clinical signs, onset, type and amount of food from cats of groups no.1–6.

Group	Clinical signs	Number of cases			Days prior to clinical signs			Only	Amount	
		total *n* = 66	acute *n* = 50	chronic *n* = 16	min	max	mean	Food I or II.	min	max
1	LMN Paraparesis	19	18	1	4	5	4.5	19	33	100
2	LMN Paraplegia	23	20	3	5	7	6	22	20	100
3	LMN Tetraparesis	11	7	4	3	10	4.5	11	25	150
4	LMN Tetraplegia	5	3	2	4	7	4.5	5	15	100
5	LMN Tetraplegia, cranial nerve deficits, and dyspnoea	5	0	5	4	10	6	5	20	100
6	Vestibular syndrome and autonomic signs	3	2	1	5	15	6	2	33	150

min: minimum; max: maximum; LMN: lower motor neuron disease; Food I and Food II: two different brands of commercial cat food contaminated with salinomycin; Amount: Amount of food expressed in percentile (100% = one can).

**Table 2:** Number of electrodiagnostic test nerve biopsies from cats of groups no.1–6.

Group	Electrodiagnostic Tests												Nerve Biopsy		
	EMG			MNCV			SNCV			RNS		
	Number of Cases			Number of Cases			Number of Cases			Number of Cases			Number of Cases		
	total	norm.	abn.	total	norm.	abn.	total	norm.	abn.	total	norm.	abn.	total	norm.	abn.
1	4	4	0	2	2	0	0	0	0	0	0	0	1	1	0
2	5	2	3	3	2	1	1	1	0	0	0	0	1	1	0
3	3	0	3	3	0	3	2	2	0	3	3	0	1	0	1
4	2	0	2	2	0	2	2	2	0	2	2	0	2	0	2
5	2	0	2	2	0	2	2	2	0	2	2	0	2	0	2
6	0	0	0	0	0	0	0	0	0	0	0	0	0	0	0
Total	16	6	10	12	4	8	8	8	0	7	7	0	7	2	5

norm.: normal; abn.: abnormal; EMG: electromyography; MNCV: motor nerve conduction velocity; SNCV: sensory nerve conduction velocity; RNS: repetitive nerve stimulation; Group 1: LMN paraparesis; Group 2: LMN paraplegia; Group 3: LMN tetraparesis; Group 4: LMN tetraplegia; Group 5: LMN tetraplegia, cranial nerve deficits and dyspnoea; Group 6: vestibular syndrome and autonomic signs.

**Table 3:** Therapy and outcome from cats of groups no. 1–6 (recovery rate and time).

Group		Therapy	Recovered							
	Total Number	Infusion Ringer-Lactated	Food I.		Food II.		Complete recovery	died	Recovery Time (Days)	
	of Cases	Penicillin	total	rec.	total	rec.			min	max
1	19	iv for 2 d	17	15	2	2	17	0	5	10
2	23	sc for 2-3 d	21	20	2	2	22	0	7	15
3	11	iv for 2-3 d	11	7	0	0	7	0	7	16
4	5	iv for 5–7 d	5	3	0	0	3	0	14	28
5	5	—	5	0	0	0	0	5	—	—
6	3	iv for 2-3 d	3	3	0	0	3	0	10	15
Total	66	—	62	48	4	4	52	5	5	28

rec.: recovered; min: minimum; max: maximum: sc: subcutaneously; iv: intravenously; Food I and Food II: two different brands of commercial cat food contaminated with salinomycin; Group 1: LMN paraparesis; Group 2: LMN paraplegia; Group 3: LMN tetraparesis; Group 4: LMN tetraplegia; Group 5: LMN tetraplegia, cranial nerve deficits, and dyspnoea; Group 6: vestibular syndrome and autonomic signs.

**Table 4:** Association between various parameters from cats of groups no. 1+2 and 3–5 and clinical scores expressed as the *P*-value.

Parameter	Groups	Groups	*P*-value
	1 + 2	3 + 4 + 5	
	combined	combined	(Fisher's Exact Test)
Acute	38	10	.0004
Chronic	4	11	
Normal blood/urin	16	3	.0011
Abnormal blood/urin	6	14	
EMG Normal	6	0	.0114
EMG Abnormal	3	7	
MNCV Normal	4	0	.0101
MNCV Abnormal	1	7	
Biopsy Normal	2	0	.0476
Biopsy Abnormal	0	5	
Recovered	39	13	.0002
Not Recovered	3	11	

Group 1: LMN paraparesis; Group 2: LMN paraplegia; Group 3: LMN tetraparesis; Group 4: LMN tetraplegia, Group 5: LMN tetraplegia; cranial nerve deficits, and dyspnoea; Group 6: vestibular syndrome and autonomic signs.
